# Sickle Cell Anemia Patients Display an Intricate Cellular and Serum Biomarker Network Highlighted by TCD4+CD69+ Lymphocytes, IL-17/MIP-1*β*, IL-12/VEGF, and IL-10/IP-10 Axis

**DOI:** 10.1155/2020/4585704

**Published:** 2020-01-08

**Authors:** Nadja Pinto Garcia, Alexander Leonardo S. Júnior, Geyse Adriana S. Soares, Thainá Cristina C. Costa, Alicia Patrine C. dos Santos, Allyson Guimarães Costa, Andréa Monteiro Tarragô, Rejane Nina Martins, Flávia do Carmo Leão Pontes, Emerson Garcia de Almeida, Erich Vinícius de Paula, Olindo Assis Martins-Filho, Adriana Malheiro

**Affiliations:** ^1^Programa de Pós-Graduação em Imunologia Básica e Aplicada, Universidade Federal do Amazonas (UFAM), 69077-000 Manaus, AM, Brazil; ^2^Laboratório de Genômica, Fundação Hospitalar de Hematologia e Hemoterapia do Amazonas (HEMOAM), 69050-001 Manaus, AM, Brazil; ^3^Programa de Pós-Graduação em Ciências Aplicadas a Hematologia, Universidade Estadual do Amazonas (PPCAH/UEA), 69065-001 Manaus, AM, Brazil; ^4^Programa de Apoio a Iniciação Científica, Fundação Hospitalar de Hematologia e Hemoterapia do Amazonas (HEMOAM), 69050-001 Manaus, AM, Brazil; ^5^Departamento de Clínica Médica da Faculdade de Ciências Médicas da UNICAMP, 13083-970 Campinas, SP, Brazil; ^6^Grupo Integrado de Pesquisas em Biomarcadores, Instituto René Rachou/Fiocruz Minas, 30190-002 Belo Horizonte, MG, Brazil

## Abstract

**Background:**

Sickle cell anemia (SCA) is associated with a chronic proinflammatory state characterized by elevated leukocyte count, mortality from severe recurrent infections, and subsequent vasoocclusive complications with leukocyte adhesion to the endothelium and increased plasma levels of inflammatory cytokines. The immune system has a close connection with morbidity in SCA, but further studies are needed to uncover the involvement of innate and adaptive immunities in modulating the SCA physiopathology. We performed measurements of the frequency of innate and adaptive immunity cells, cytokines, chemokines, and growth factors and immunophenotyping of Toll-like receptor and adhesion molecule expression in the blood of SCA patients and healthy donors to evaluate the different profiles of these biomarkers, the relationship among them, and their correlation to laboratory records and death risk. *Material and Methods*. Immunophenotyping of cells, Toll-like receptors, and adhesion molecules were performed from peripheral blood samples of SCA patients and healthy donors by flow cytometry and cytokine/chemokine/growth factor measurement by the Luminex technique performed from the serum of the same subjects.

**Results:**

Cells of adaptive immunity such as IL-12, IL-17, and IL-10 cytokines; IL-8, IP-10, MIP-1*α*, MIP-1*β*, and RANTES chemokines; and VEGF, FGF-basic, and GM-CSF growth factors were higher in SCA patients than healthy donors regardless of any laboratorial and clinical condition. However, high death risk appears to have relevant biomarkers.

**Conclusion:**

In the SCA pathophysiology at steady state, there is a broad immunological biomarker crosstalk highlighted by TCD4+CD69+ lymphocytes, IL-12 and IL-17 inflammatory and IL-10 regulatory cytokines, MIP-1*α*, MIP-1*β*, and IP-10 chemokines, and VEGF growth factor. High expression of TLR2 in monocytes and VLA-4 in TCD8+ lymphocytes and high levels of MIP-1*β* and RANTES appear to be relevant in high death risk conditions. The high reticulocytosis and high death risk conditions present common correlations, and there seems to be a balance by the Th2 profile.

## 1. Introduction

Sickle cell anemia is the most common hemoglobinopathy (>70% of sickle cell disease in the world) and the most severe form resulting from homozygous inheritance; a point mutation of adenine is replaced by thymine (GAG → GTG) in the sixth codon of the *β*-globin gene (*β*S), with valine replacing glutamic acid at the sixth position of the polypeptide chain reflecting as an abnormal form of hemoglobin (HbS) [1–3].

It is estimated that each year, 300,000 children are born with a severe form (homozygotes) worldwide, mainly in sub-Saharan Africa, the Middle East, and India. Migration patterns led to the distribution of the sickle cell gene to nonendemic areas of malaria, such as areas in Europe and the USA. In Brazil in 2016, 1,071 newborn babies had sickle cell disease (SCD) and >60,000 were heterozygous for the *β*^S^ allele. There are an estimated 30,000 individuals with SCD in the whole country [4–6], and the prevalence of heterozygotes for HbS in the North and Northeast regions is higher at about 6 to 10%, while in the South and Southeast, the prevalence is lower at 2 to 3% [7, 8].

Sickle cell anemia is associated with a chronic proinflammatory state characterized by an elevated leukocyte count, mortality from severe recurrent infections, and subsequent vasoocclusive complications such as leukocyte adhesion to the endothelium and increased plasma levels of inflammatory cytokines [9, 10]. Although patients have the same genetic mutation for HbS, SCA is characterized by a very heterogeneous clinical course, ranging from patients who have normal life expectancy with relatively few complications such as pulmonary hypertension, priapism, stroke, leg ulceration, recurrent painful episodes, acute chest syndrome (ACS), and avascular necrosis of bone [11–13]. This variability can partly be explained by genetic modifiers, including factors that affect HbF level and coinheritance of *α*-thalassaemia or by environmental factors [9, 13–15]. The immune system has a close connection with morbidity in sickle cell anemia, but further studies are needed to uncover the involvement of innate and adaptive immunities in modulating the SCA physiopathology [9, 14].

The main events of the SCA physiopathology are hemolysis and vasoocclusive painful crises due to the complex phenomenon of HbS polymerization essential for the occurrence of SCA. This phenomenon alters the typical lipid bilayer and proteins of the erythrocyte membrane, which leads to a reduction of cellular hydration and changes in the shape and physical properties of erythrocytes. All these changes will result in hemolytic anemia, abnormal interactions with other blood cells, early erythrocyte apoptosis, and blockage of blood flow, particularly in small (and some large) vessels, which lead to repeated hypoxia-reperfusion (oxygenation-deoxygenation) processes further damaging any other organ [16, 17]. The highly unstable sickle erythrocytes reduce lifespan to ≥75% which induces an increased response of bone marrow and therefore increased erythropoiesis, reflected by the high circulation of reticulocytes, which account for ~20% of the red blood cells in individuals with SCA [15, 18]. Hemolysis is thought to occur principally via extravascular phagocytosis by macrophages, but a substantial fraction occurs via intravascular hemolysis [15, 19]. The release of erythrocyte content into plasma by intravascular hemolysis interferes incisively on vasculopathy with the involvement of coagulation and immune system components [15].

Vasoconstriction and vascular remodeling in SCA patients, especially in the lung, are mainly caused by nitric oxide (NO) depletion. The decrease of NO bioavailability is caused by Hb (hemoglobin) that scavenges NO, enzyme NO synthase (NOS) inhibition by asymmetric dimethylarginine (ADMA), and by L-arginine depletion, the substrate of NOS, by arginase 1 enzyme. The NO deficiency promotes platelet activation and activation of blood clotting proteins [15, 20].

Heme and other danger-associated molecular pattern (DAMP) molecules released from erythrocytes activated the innate immune system. Ligand-bound Toll-like receptor 4 (TLR-4) and TLR-2 activate monocytes and macrophages to release inflammatory cytokines, which promote an inflammatory state and activation of endothelial cells. TLR-4 activation on platelets promotes their adhesion to neutrophils, which in turn release DNA to form neutrophil extracellular traps (NETs). Circulating blood cells adhere to each other and to the activated endothelium, contributing and potentially even initiating vasoocclusion [21–23].

Limited studies conducted thus far have indicated adaptive abnormalities in individuals with SCA. Some abnormalities like reduction in the proportion of circulating CD4+ and CD8+ T-cells and dysfunction of regulatory T-cells occur concurrent with increased immune activation and may affect vaccine reactivity in individuals with SCA [24–27]. It is possible that geographical differences in the immune system function may also occur between SCA populations [27].

Considering the divergent information about the complex immune response on the progression of SCA, in this study we proposed to explore the role of immunological biomarkers during SCA steady state. So, we performed measurements of the frequency of innate and adaptive immunity cells, cytokines, chemokines, and growth factors and immunophenotyping of Toll-like receptors and adhesion molecule expression in the blood of SCA patients and healthy donors to evaluate the different profiles of these biomarkers, the relationship among them, and their correlation to laboratory records and death risk.

## 2. Subjects, Material, and Methods

### 2.1. Ethics Statement

All protocols and consent forms were approved by the Ethical Committees on Research from the Fundação Hospitalar de Hematologia e Hemoterapia do Amazonas (HEMOAM) through the protocol CAEE (no. 56413316.9.0000.0009). The subjects involved in the study signed an informed written consent form in accordance to Resolution 196/96 of the National Health Board for research involving human subjects prior to participating.

### 2.2. Study Population

This study included 100 subjects categorized into the following two subgroups: the sickle cell anemia (SCA) patient group with 30 subjects at steady state, consisting of patients who were not in crises and had not received blood transfusions in the preceding 3 months and the healthy donor (HD) group with 70 subjects without any apparent disease. A nonprobabilistic convenience sampling was performed to select patients and healthy donors that go to the Fundação Hospitalar de Hematologia e Hemoterapia do Amazonas (HEMOAM, Manaus, Amazonas, Brazil) for treatment and blood donation, respectively, with negative results for serologic screening tests, including viral hepatitis (A, B, and C), HIV, syphilis, Chagas disease, and HTLV-1/2. The SCA patients' group was composed of subjects of both genders' (20 females and 10 males), with ages ranging from 18 to 49 years (30 ± 8.9), and homozygous to HbS (HbSS). The HD group was composed of subjects of both genders' (52 males and 18 females), with ages ranging from 20 to 61 years (32 ± 10.6). Pregnant women, indigenous people, subjects with other hematological diseases (leukemia and other anemias), subjects undergoing or have been treated for cancer, and subjects with inflammatory and infectious diseases such as malaria, dengue, Chagas Disease, mycoses, Zika, Chikungunya, pneumonia, pharyngitis, tonsillitis, and bronchitis, were not included.

The choice of SCA subgroups was based on the clinical importance of hemolysis (reticulocytes), hemostasis, and inflammation (platelets) and clinical complications inherent to the physiopathology of sickle cell anemia. The association of reticulocytosis and thrombocytosis (platelet count above 400 × 10^6^/mm^3^) in the clinical severity of this disease was shown in previous studies [28]. The categorizations of these subgroups were done by GraphPad Prism version 5.0 software (San Diego, CA, USA) using descriptive analysis establishing the minimum 25% percentile, the median, and the maximum 75% percentile. Considering that all patients present reticulocytosis and thrombocytosis, we classify them as low (below the median) and high (above the median). The median of reticulocytes was 400 × 10^6^/mm^3^ and that of platelets was 450 × 10^6^/mm^3^. The death risk as calculated by the “Sickle Cell Disease Severity Calculator,” available from http://www.bu.edu/sicklecell/downloads/Projects, is described previously for the calculation of severity scores and the classification of patients into categories by phenotype (mild, intermediate, and severe) [13]. However, we did not use the rating used by Junior et al. in 2015 because the patient population would be classified as 4/30 as intermediate and 26/30 as severe, which would make it impossible to segregate patients by robust statistical analysis. Therefore, the criterion of the median severity score (0.670, 67%) was chosen to classify the patients as more and less severe, with 15/30 with low severity (<0.670) and 15/30 with high severity (>0.670).

The clinical and demographical data were acquired by a standardized questionnaire, and the hematological profile was assessed by automated blood count carried out at Fundação Hospitalar de Hematologia e Hemoterapia do Amazonas. The demographic data and hematological and clinical characteristics of patients and healthy donors are summarized in Table 1.

### 2.3. Immunophenotypic Analysis of Innate and Adaptive Components

The immunophenotypic characterization was performed by a flow cytometry technique. The cells were obtained from an aliquot of 100 *μ*L from peripheral blood collected with EDTA and were incubated in the presence of the following fluorescent-labeled specific anti-human cell surface monoclonal antibodies: anti-CD3FITC/CD8PE/CD4PercP/CD69APC to identify CD4+ and CD8+ T-cells; anti-CD4FITC/CD25PercP/FoxP3 AF647 to identify regulatory T-cells; anti-CD5FITC/CD19PE to identify B and B1 cells; anti-CD16FITC/CD56PE/CD3PercP/CD69APC to identify NK and NKT cells; anti-CD123FITC/CD11cPE to identify classical and plasmacytoid dendritic cells; anti-CD14FITC/CD80PE/CD16PercP/HLA-DRAPC to identify monocyte subtypes; anti-CD14FITC/CD16PercP/CD282PE TLR2 to identify TLR2 expression in monocytes and neutrophils; anti-CD14FITC/CD16PercP/CD284PE to identify TLR4 expression in monocytes and neutrophils; anti-CD14FITC/CD16PercP/CD289PE to identify TLR9 expression in monocytes and neutrophils; anti-CD4FITC/CD11bPE/CD3PercP to identify Mac-1 expression in CD4+ T-cells; anti-CD8FITC/CD11bPE/CD3PercP to identify Mac-1 expression in CD8+ T-cells; anti-CD4FITC/CD49dPE/CD3PercP to identify VLA-4 expression in CD4+ T-cells; and anti-CD8FITC/CD49dPE/CD3PercP to identify VLA-4 expression in CD8+ T-cells (antibodies were purchased from BD Biosciences (San Diego, CA, USA), Beckman Coulter (Brea, California, USA), and BioLegend (San Diego, CA, USA)). Following incubation, cells were treated with 2 mL of erythrocyte lysing solution for 7.5 min at room temperature. After two centrifugation steps and one wash step with PBS, nonintracellularly labeled cells were resuspended in 300 *μ*L PBS and intracellularly labeled cells were incubated with PBS-saponin, followed by a centrifugation step and resuspension in PBS. Stained cells were stored at 4°C up to 24 h prior to flow cytometric acquisition. A total of 30,000/100,000 events were acquired for each blood sample to quantify cell types.

The sample acquisition was performed on the FACSCalibur® Flow Cytometer (Becton, Dickinson and Company, San Jose, CA, USA) of the Fundação HEMOAM. For the morphometric and immunophenotypic identification of the cells, the FlowJo Software (version 7.2) was used, with the design of the gates to select the target populations in graphs that combine morphological characteristics (size and granulosity) with immunophenotypic characteristics through the fluorescence of the monoclonal antibodies used to identify the target cells.

### 2.4. Cytokines, Chemokines, and Growth Factor Analysis

The serum cytokines IL-1*β*, IL-6, TNF-*α*, IL-12, IFN-*γ*, IL-2, IL-7, IL-4, IL-5, IL-13, IL-17, and IL-10; chemokines IL-8, IP-10, MIP-1*α*, MIP-1*β*, MCP-1, and RANTES; and growth factors VEGF, FGF-basic, PDGF, GM-CSF, and G-CSF were measured using Bio-Plex Pro Human Cytokine 27-Plex Kit (Bio-Rad, California, USA) following the manufacturer's instructions by Luminex assay. Data acquisition and analysis were performed using Luminex 200 System equipment and Bio-Plex Manager Software, respectively. The results were expressed as pg/mL, as assessed by the standard curve using the fifth-logistic regression parameter. The limits of detection were IL − 1*β* = 8.608 pg/mL, IL − 6 = 37.680 pg/mL, TNF − *α* = 64.803 pg/mL, IL − 12 = 37.684 pg/mL, IFN − *γ* = 25.411 pg/mL, IL − 2 = 18.297 pg/mL, IL − 7 = 16.593 pg/mL, IL − 4 = 4.789 pg/mL, IL − 5 = 23.105 pg/mL, IL − 13 = 8.090 pg/mL, IL − 17 = 28.850 pg/mL, IL − 10 = 35.170 pg/mL, IL − 8 = 42.150 pg/mL, IP − 10 = 31.236 pg/mL, MIP − 1*α* = 960 pg/mL, MIP − 1*β* = 11.233 pg/mL, MCP − 1 = 24.282 pg/mL, RANTES = 16.533 pg/mL, VEGF = 29.464 pg/mL, FGF − basic = 16.046 pg/mL, PDGF = 24.721 pg/mL, GM − CSF = 12.844 pg/mL, and G − CSF = 40.049 pg/mL.

### 2.5. Data Analysis and Conventional Statistics

All data were considered as presenting a nonparametric distribution, and therefore, the comparative analyses about the frequency of cells and levels of cytokines, chemokines, and growth factors were compared between HD and SCA groups by the Mann-Whitney two-tailed test. Analyses between the low and high subgroups were performed using the ANOVA variance analysis, followed by the Kruskal-Wallis test, and followed by Dunn's multiple comparison test. A 95% confidence interval was used, and the data considered with statistical significance were those with *p* value < 0.05. The GraphPad Prism software version 5.0 (San Diego, CA, USA) was used for data analysis.

### 2.6. Biomarker Signature Analysis

The cellular and serum biomarker ascendant signatures were assembled as previously reported by Luiza-Silva et al. [29]. This model of analysis allows converting continuous measurements into a categorical analysis. Initially, the whole universe of data of each biomarker was used to calculate the global median value used as the cut-off to classify each subject as the present values below or above the cut-off edge. Thereafter, the ascendant signatures of the cell phenotype features and serum immunological biomarkers were assembled considering the frequency of subjects with values above the global median cut-off determined for each biomarker. Overlays of ascendant biomarker signature curves were employed to identify those biomarkers with the frequency of subjects above the 50th percentile, further highlighted for subsequent Venn diagram analysis to identify those biomarkers commonly or selectively observed among groups. The GraphPad Prism 5.0 software (San Diego, USA) was used for graph arts.

### 2.7. Biomarker Network Assembly

Biomarker networks were assembled to evaluate the multiple associations among the cells and cytokines/chemokines/growth factors in the SCA patients and subgroups. The association between the quantitative levels of cells, cytokines, chemokines, and growth factors were determined by the Spearman correlation coefficient in GraphPad Prism 5.0 software (San Diego, USA), and statistical significance was considered only if *p* < 0.05. After performing the correlation analysis between biomarkers, a database was created on the Microsoft Excel program 2010. Then, the significant correlations were compiled using the open access software Cytoscape (version 3.6.1) as previously reported. The biomarker networks were constructed using circular nodes for each cell, cytokine, chemokine, and growth factor, including white circles to HD, black circles to SCA, light grey circles to low subgroups, and dark grey circles to high subgroups assembled in circular layouts. The correlation indices (*r*) were used to categorize the correlation strength as negative (*r* < 0), moderate (0.36 ≥ *r* ≤ 0.68), and strong (*r* > 0.68) represented by connecting edges as proposed by Taylor [30].

## 3. Results

### 3.1. Major Phenotypic Features of Innate and Adaptive Immunity in Patients with Sickle Cell Anemia

**Figure 1:**
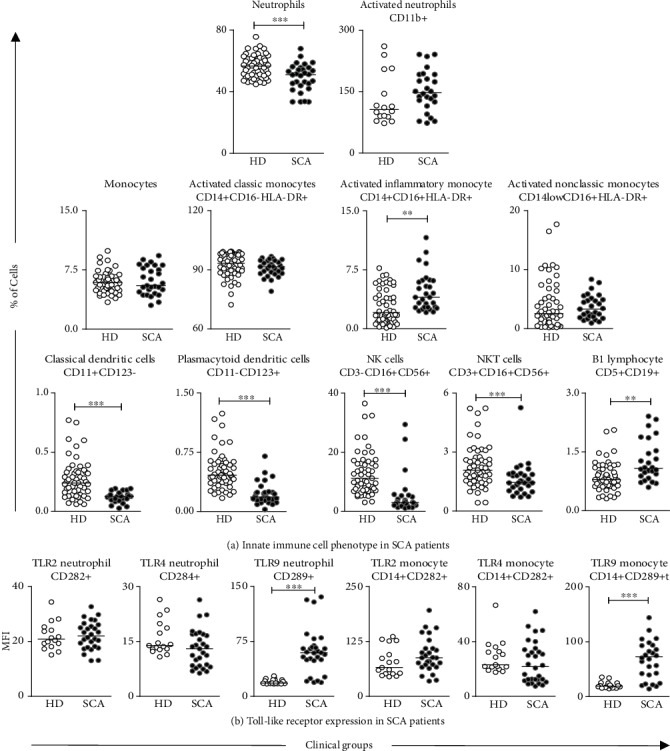
Phenotype profile of innate immunity in SCA patients. The frequency of cell phenotypes (a) and Toll-like receptor expression (b) were measured in peripheral blood of SCA patients and healthy donors by flow cytometry. The results are shown in scatter plots with individual values, and the median percentage of cells or mean fluorescence intensity (MFI) is represented as a line. Significant differences (*p* < 0.05) are highlighted by connecting lines and asterisks (∗) for comparison with the HD group.

**Figure 2:**
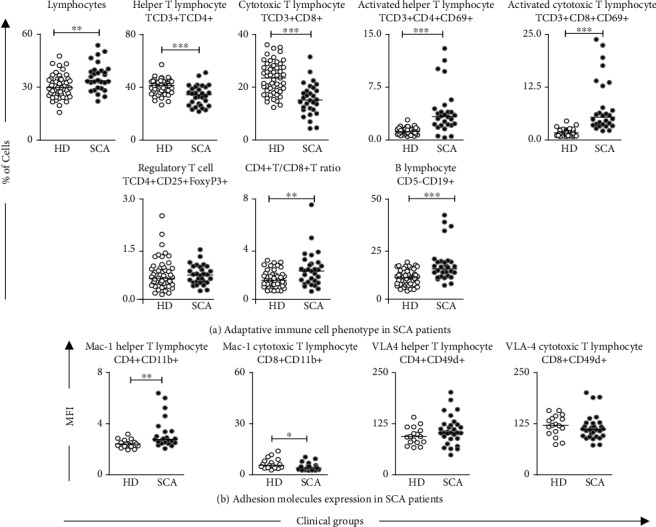
Phenotype profile of adaptive immunity in SCA patients. The frequency of cell phenotypes (a) and adhesion molecule expression (b) were measured in peripheral blood of SCA patients and healthy donors by flow cytometry. The results are shown in scatter plots with individual values, and the median percentage of cells or mean fluorescence intensity (MFI) is represented as a line. Significant differences (*p* < 0.05) are highlighted by connecting lines and asterisks (∗) for comparison with the HD group.

The innate immunity cell phenotypes in SCA patients are presented in Figure 1. The low frequency of neutrophils and classical and plasmacytoid DCs, along with the decreased percentage of NK and NKT cells, were observed in SCA as compared to HD (Figure 1(a)). Conversely, the increased frequency of activated inflammatory monocytes (CD14+CD16+HLA-DR+) and B1 lymphocytes as well as the enhanced expression of TLR-9 by neutrophils and monocytes were also observed in SCA patients (Figures 1(a) and 1(b)). Phenotypic features of adaptive immunity cells are presented in Figure 2. Data analyses demonstrated the high frequency of lymphocytes, activated TCD4+, TCD8+, TCD4+/TCD8+ ratio, and B lymphocytes and the high expression of Mac-1 in TCD4+ cells in SCA patients compared to HD (Figures 2(a) and 2(b)). Furthermore, decreased levels of CD4+ and CD8+ lymphocytes, besides the decreased expression of Mac-1 in TCD8+ cells were also observed in the SCA group (Figures 2(a) and 2(b)).

### 3.2. Phenotypic Features of Innate and Adaptive Immunity in Patients with Sickle Cell Anemia according to Laboratorial and Clinical Records

**Figure 3:**
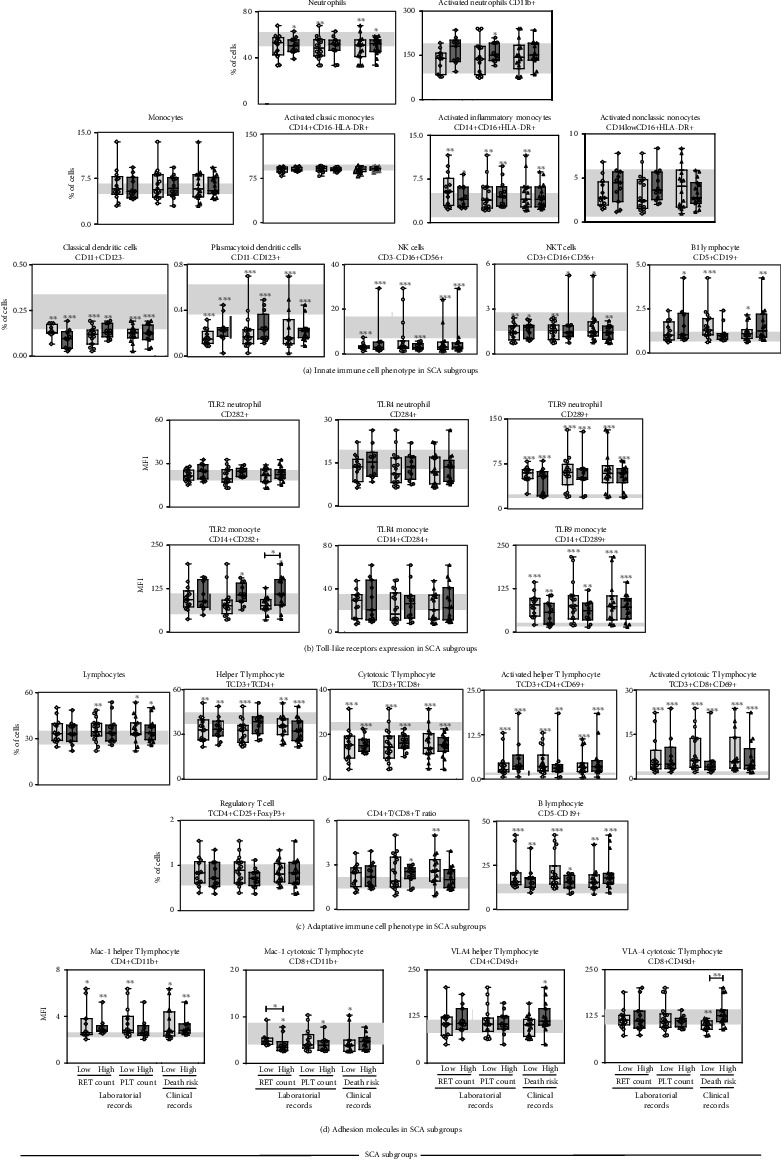
Phenotype profile of innate and adaptive immunity components in SCA subgroups according to laboratorial and clinical records. The frequency of cell phenotypes (a), the expression of Toll-like receptors (b) by innate immunity cells along with the cell phenotypes (c), and the expression of adhesion molecules by adaptive immunity cells (d) were analyzed in SCA subgroups by flow cytometry. The SCA patients were categorized according to their laboratorial and clinical records, including reticulocyte counts, platelet levels, and death risk scores. The results are presented in box plots with median and interquartile range overlaid by scatter plots with individual values for cell phenotype or mean fluorescence intensity (MFI). The interquartile ranges (25th-75th) for results observed in HD were used as reference range (gray background). Significant differences (*p* < 0.05) are highlighted by connecting lines and asterisks (∗).

Aiming at further characterizing the profile of innate and adaptive immunity cells in SCA, the patients were categorized according to their laboratorial and clinical records, including reticulocyte counts, platelet levels, and death risk scores. The results are presented in Figure 3. The reference values observed in HD are presented as the interquartile range (25th-75th) for each cell phenotype evaluated (Figure 3, gray background). Data analysis demonstrated that the high frequency of activated inflammatory monocytes (CD14+CD16+HLA-DR+) and increased expression of TLR9 by neutrophils and monocytes are observed in SCA patients regardless of the laboratorial and clinical conditions. Moreover, lower levels of classical and plasmacytoid DCs, besides NK and NKT cells, were also found in SCA patients regardless of the presence of reticulocytosis, thrombocytosis, and high death risk (Figures 3(a) and 3(b)). The SCA patients with a high platelet count showed an elevated frequency of activated neutrophils and increased expression of TLR2 by monocytes compared to HD (Figures 3(a) and 3(b)). Furthermore, the enhanced expression of TLR2 by monocytes was observed in SCA patients with a high death risk score (Figure 3(b)).

The analysis of adaptive immunity cells revealed that despite the low levels of CD4+ T-cells and CD8+ T-cells, SCA patients exhibited a high frequency of CD4+CD69+ T-cells, CD8+CD69+ T-cells, and CD19+ B-cells regardless of the laboratorial/clinical score (Figure 3(c)). In addition, the results demonstrated that Mac-1 expression by CD8+ T-cells was significantly lower in SCA patients with low reticulocyte counts (Figure 3(d)). Moreover, increased VLA-4 expression by CD8+ T-cells was found in SCA with a high death risk score (Figure 3(d)). Remarkably, SCA patients with high death risk presented an increased expression of TLR2 by monocyte and VLA-4 by CD8+ T-cells in comparison with those SCA patients presenting low death risk (Figures 3(b) and 3(d)). Conversely, SCA patients with low reticulocyte counts showed decreased expression of Mac-1 by CD8+ T-cells as compared to patients with high reticulocyte counts (Figure 3(d)).

### 3.3. Serum Immunological Biomarkers in Sickle Cell Anemia Patients

**Figure 4:**
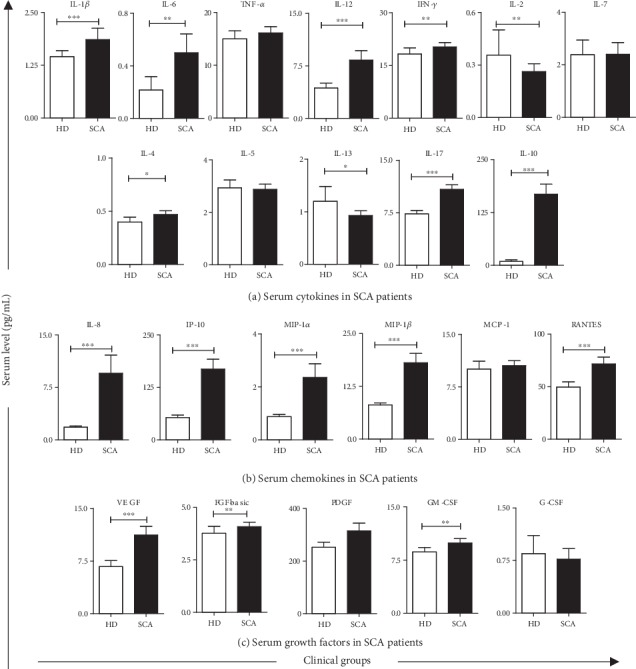
Serum immunological biomarkers in sickle cell anemia patients. The levels of cytokines (a), chemokines (b), and growth factors (c) were measured in sera of SCA patients and healthy donors by Luminex assay. The results are shown in bar charts with mean ± standard deviation for serum concentration (pg/mL). Significant differences (*p* < 0.05) are highlighted by connecting lines and asterisks (∗).

The profile of serum cytokines, chemokines, and growth factors observed in SCA patients are shown in Figure 4. Data demonstrated that SCA patients displayed an overall increase of most immunological biomarkers evaluated, include IL-1*β*, IL-6, IL-12, IFN-*γ*, IL-4, IL-17, IL-10, IL-8, IP-10, MIP-1*α*, MIP-1*β*, RANTES, VEGF, FGF-basic, and GM-CSF as compared to HD (Figures 4(a)–4(c)). Decreased levels of IL-2 and IL-13 were also observed in SCA patients (Figure 4(a)). Unaltered levels of TNF-*α*, IL-7, IL-5, MCP-1, and G-CSF were found in serum samples from SCA patients as compared to HD (Figures 4(a)–4(c)).

### 3.4. Serum Immunological Biomarkers in Patients with Sickle Cell Anemia according to Laboratorial and Clinical Records

**Figure 5:**
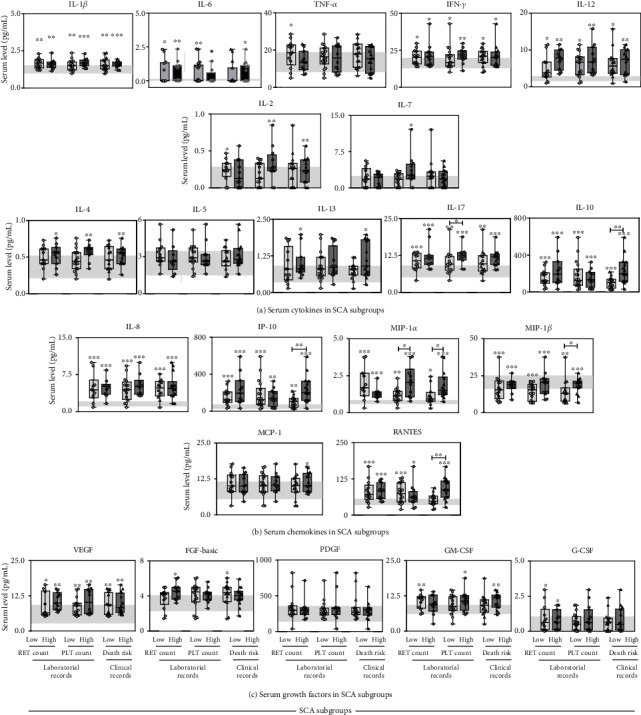
Serum immunological biomarkers in sickle cell anemia patients according to laboratorial and clinical records. The levels of cytokines (a), chemokines (b), and growth factors (c) were measured in sera of SCA subgroups by Luminex assay. The SCA patients were categorized according to their laboratorial and clinical records, including reticulocyte counts, platelet levels, and death risk scores. The results are presented in box plots with median and interquartile range overlaid by scatter plots with individual serum concentration (pg/mL). The interquartile ranges (25th-75th) for results observed in HD were used as reference range (gray background). Significant differences (*p* < 0.05) are highlighted by connecting lines and asterisks (∗).

The serum levels of cytokines, chemokines, and growth factors were further evaluated in SCA patients categorized according to their laboratorial and clinical records, and data are shown in Figure 5. The reference values observed in HD are presented as the interquartile range (25th-75th) for each immunological biomarker analyzed (Figure 5, gray background). Increased levels of IL-1*β*, IFN-*γ*, IL-12, IL-17, IL-10, IL-8, IP-10, MIP-1*α*, and VEGF but low levels of MIP-1*β* were observed in SCA patients regardless of the laboratorial and clinical scores as compared to HD reference ranges. The high concentrations of IL-6, RANTES and G-CSF were shown in SCA patients regardless of the reticulocyte counts. TNF-*α*, IL-2, and GM-CSF were increased in SCA patients with low reticulocyte counts. IL-4, IL-13, and FGF-basic were detected in SCA patients with high reticulocyte counts as compared to HD reference ranges. High levels of IL-6 were demonstrated in SCA patients with low platelet counts. Enhanced levels of IL-2, IL-7, IL-4, and GM-CSF were observed in SCA patients with high platelet counts as compared to HD reference ranges. Increased levels of IL-6, IL-4, IL-13, MCP-1, and GM-CSF were found in SCA patients with high death risk as compared to HD reference ranges. Remarkably, SCA patients with high death risk presented increased levels of IL-10, IP-10, MIP-1*α*, MIP-1*β*, and RANTES in comparison with those of SCA patients presenting low death risk. Likewise, SCA patients with high platelet counts presented increased levels of IL-17 and MIP-1*α* as compared to those with low platelet counts (Figures 5(a)–5(c)).

### 3.5. Signatures of Cell Phenotype Features and Serum Immunological Biomarkers in Patients with Sickle Cell Anemia according to Laboratorial and Clinical Records

**Figure 6:**
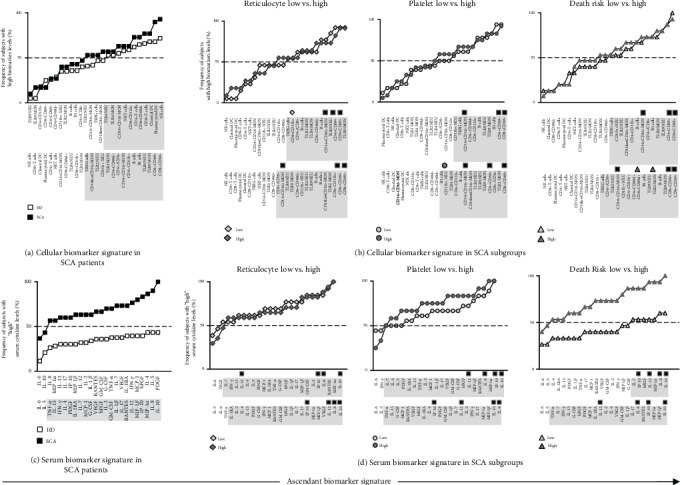
Signatures of cell phenotype features and serum immunological biomarkers in patients with sickle cell anemia according to laboratorial and clinical records. The signatures of cell phenotype features (a, b) and serum immunological biomarkers (c, d) were assembled considering the frequency of subjects with values above the global median cut-off determined for each biomarker. The SCA patients were categorized according to their laboratorial and clinical records, including reticulocyte counts, platelet levels, and death risk scores. The global median values for each biomarker were used as the cut-off to classify each subject with “low” or “high” biomarker levels. Overlay of ascendant biomarker signature curves was employed to identify those biomarkers with frequency of subjects above the 50th percentile (dashed lines) further highlighted by gray background. Venn diagram analyses were carried out to identify those biomarkers commonly or selectively observed among groups. The universal biomarkers observed in all SCA subgroups were tagged by black squares. Those biomarkers with putative association with SCA subgroups were tagged with a gray diamond for SCA patients with low reticulocyte counts, a dark gray circle for SCA patients with high platelet counts, or a dark gray triangle for SCA patients with high death risk.

The signatures of cell phenotype features and serum immunological biomarkers were assembled as the frequency of subjects with values of cells from innate and adaptive immunity or serum biomarkers above the global median cut-off, aiming at identifying the attributes that characterize the immunological profile of SCA patients and their respective laboratorial and clinical records. According to this categorical descriptive analysis, it was possible to select the most relevant biomarkers observed in more than 50% of the subjects on each group (Figure 6—gray background). Data analysis demonstrated that in general the SCA patients presented higher number biomarkers above the 50th percentile as compared to HD (Figure 6(a)). Slight differences were observed in the number of cellular biomarkers above the 50th percentile while comparing the SCA subgroups (Figure 6(b)).

Venn diagram analyses further demonstrated that a set of cell phenotype features were commonly observed in more than 50% of the SCA patients, regardless of their laboratorial and clinical records, including CD14+CD16+MON, CD4+CD69+, and CD8+CD69+ (Figure 6(b)—black squares). Furthermore, specific biomarkers were selectively identified in the ascendant signatures of SCA patients with low reticulocyte counts, high platelet counts, and high death risk, including CD8+CD11b+, CD11b+NEU, CD8+CD49d+, and TLR2MON, respectively (Figure 6(b)).

A clear cytokine storm was observed in SCA patients as compared to HD. Data analysis demonstrated that SCA patients presented a massive increase in most serum immunological biomarkers as observed by the frequency of subjects with levels above the 50th percentile as compared to HD (Figure 6(c)). Comparative analysis between SCA subgroups demonstrated that SCA patients with a high death risk display a higher number of altered biomarkers as compared to those with a low death risk (Figure 6(d)). Venn diagram analyses demonstrated that a set of serum biomarkers were commonly observed in more than 50% of the SCA patients, regardless of their laboratorial and clinical records, including IL-8, IP-10, IL-12, and IL-10 (Figure 6(d)—black squares). However, no selective biomarkers could be identified for a given SCA subgroup (Figure 6(d)).

### 3.6. Cell Phenotypes and Serum Biomarker Networks in Patients with Sickle Cell Anemia according to Laboratorial and Clinical Records

**Figure 7:**
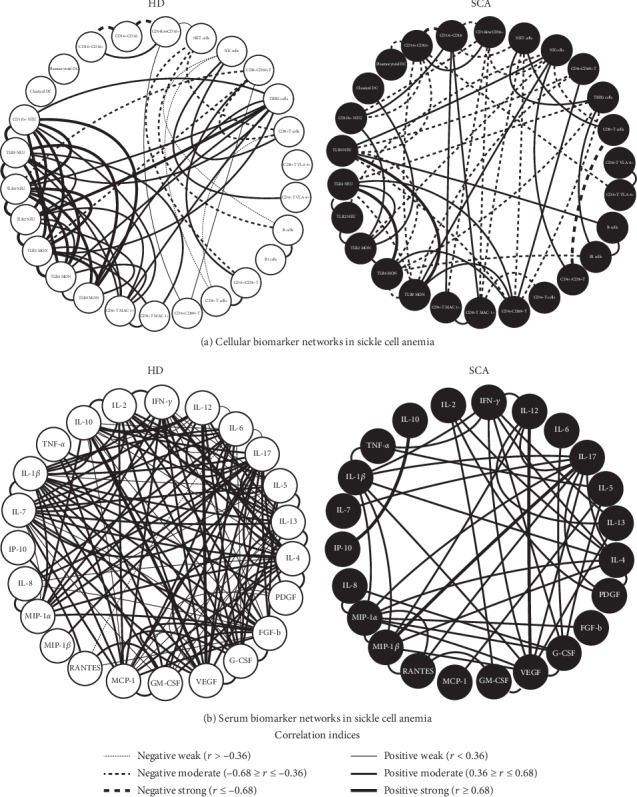
Cellular and serum biomarker networks in SCA patients. Customized biomarker network layouts for SCA patients (black nodes) and HD (white nodes) were assembled to identify the relevant association between innate and adaptive cell phenotypes (a) and serum biomarkers (b) using circular distribution of nodes. Significant Spearman's correlations at *p* < 0.05 were represented by connecting edges to highlight positive (strong (*r* ≥ 0.68; thick continuous line), moderate (0.36 ≥ *r* ≤ 0.68; not so thick continuous line), or weak (*r* < 0.36; thin continuous line)) and negative (strong (*r*≤−0.68; thick dashed line), moderate (−0.68 ≥ *r*≤−0.36; not so thick dashed line), or weak (*r*>−0.36; thin dashed line)) as proposed by Taylor [30].

**Figure 8:**
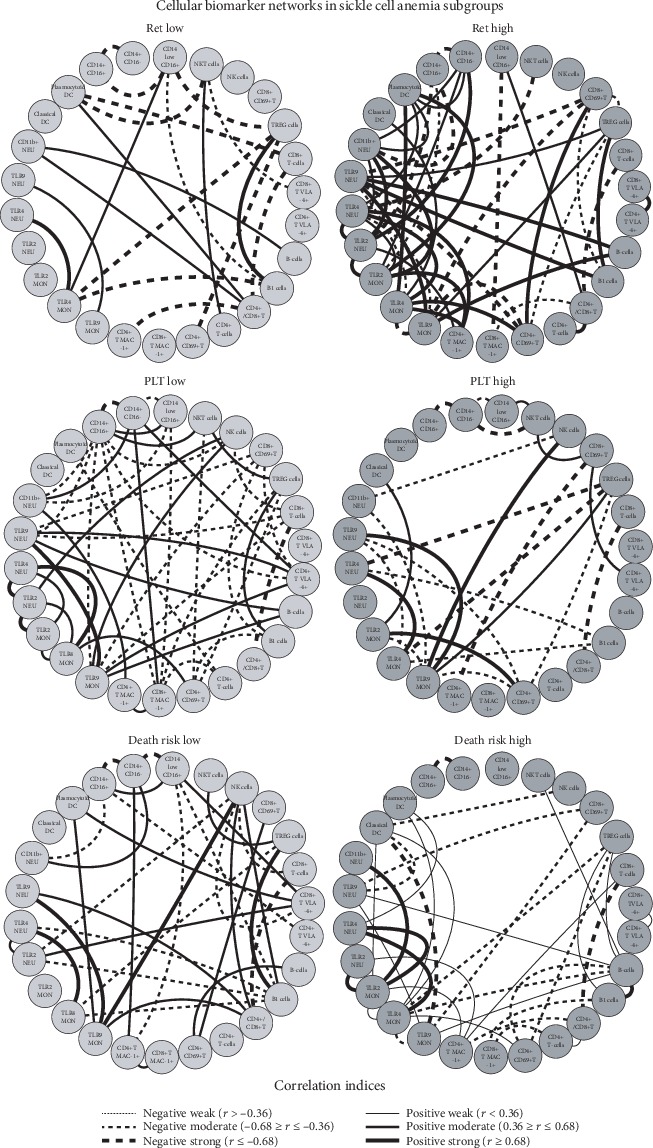
Cell phenotype networks in patients with sickle cell anemia according to laboratorial and clinical records. The SCA patients were categorized according to their laboratorial and clinical records, including reticulocyte counts (RET), platelet levels (PLT), and death risk scores. Customized biomarker network layouts were assembled to identify the relevant association between innate and adaptive cell phenotypes for SCA patients with low (light gray nodes) or high (dark gray nodes) levels. Clinical and laboratorial records were assembled to identify the relevant association between innate and adaptive cells using the circular distribution of nodes. Significant Spearman's correlations at *p* < 0.05 were represented by connecting edges to highlight positive (strong (*r* ≥ 0.68; thick continuous line), moderate (0.36 ≥ *r* ≤ 0.68; not so thick continuous line), or weak (*r* < 0.36; thin continuous line)) and negative (strong (*r*≤−0.68; thick dashed line), moderate (−0.68 ≥ *r*≤−0.36; not so thick dashed line), or weak (*r*>−0.36; thin dashed line)) as proposed by Taylor [30].

**Figure 9:**
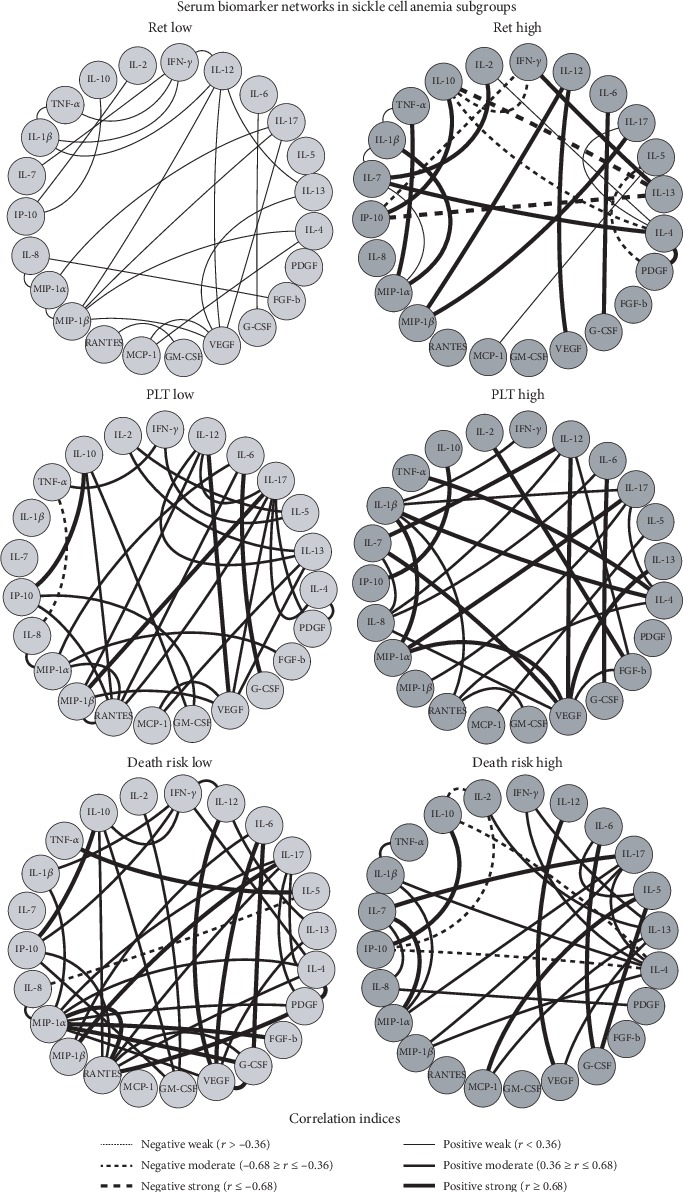
Serum biomarker networks in patients with sickle cell anemia according to laboratorial and clinical records. The SCA patients were categorized according to their laboratorial and clinical records, including reticulocyte counts (RET), platelet levels (PLT), and death risk scores. Customized biomarker network layouts were assembled to identify the relevant association between innate and adaptive cell phenotypes for SCA patients with low (light gray nodes) or high (dark gray nodes) levels. Clinical and laboratorial records were assembled to identify the relevant association between serum cytokines, chemokines, and growth factors using circular distribution of nodes. Significant Spearman's correlations at *p* < 0.05 were represented by connecting edges to highlight positive (strong (*r* ≥ 0.68; thick continuous line), moderate (0.36 ≥ *r* ≤ 0.68; not so thick continuous line), or weak (*r* < 0.36; thin continuous line)) and negative (strong (*r*≤−0.68; thick dashed line), moderate (−0.68 ≥ *r*≤−0.36; not so thick dashed line), or weak (*r*>−0.36; thin dashed line)) as proposed by Taylor [30].

Aiming at characterizing the integrative and multiparametric analysis of the immune response in SCA patients, a correlation analysis between cell phenotypes as well as among serum biomarkers was carried out and the network profiles assembled according to the negative or positive correlations between pairs of attributes (Figures 7, 8, and 9). Data demonstrated that SCA patients presented a complex and intricate cell phenotype network rich in negative connections compared to HD (Figure 7(a)). In fact, while HD displayed a strong correlation between innate immunity receptors and Treg cells, the SCA patients displayed positive connections between CD4+CD69+T and CD8+CD69+, TLR4NEU, TLR4MON, and TLR9MON along with others involving TLR4NEU, TLR2NEU, TLR4MON, and TLR2MON.

Analysis of serum biomarker networks revealed that a small number of positive edges were observed in SCA patients as compared to HD (Figure 7(b)). It was noticed that while in HD, IL-10 has several connections modulating the immune response and made an interesting triad between IL-10, IL-12, and VEGF, whereas in SCA patients, IL-10 displayed a relevant loss of connections with only a strong positive connection with IP-10 remaining, which was not observed in HD. In SCA, interesting triads are observed, including IFN-*γ*/IL-12/VEGF, IL-12/IL-17/MIP-1*β*, and IL-1*β*/IFN-*γ*/TNF-*α*, suggesting an intense inflammatory response.

The cellular networks assembled for SCA subgroups according to laboratory and clinical records are presented in Figure 8. The results demonstrated that SCA patients with high reticulocyte counts (RET high subgroup) presented a stronger network compared to those with low reticulocyte counts (RET low subgroup). On the other hand, SCA patients with high platelet counts or high death risk presented a significant loss of connection. Interestingly, patients with high reticulocyte counts and high death risk presented strong connections such as TLR9NEU/B-cell/TLR9MON and CD4+CD69+T/TLR4MON. The SCA subgroup with low platelet counts has a peculiar network with a relevant increase of negative connections (Figure 8).

The serum biomarker network assembled for SCA subgroups according to laboratory and clinical records are presented in Figure 9. Data analysis demonstrated that in general, SCA patients presented positive connections between the pairs IL-17/MIP-1*β*, IL-12/VEGF, and IL-10/IP10, regardless of their laboratorial records. It was noticed that although IL-10 makes few connections, the IL-10/IP-10 link is observed in all SCA subgroups. SCA subgroups with high reticulocyte counts presented stronger connections as compared to those with low reticulocyte counts. However, SCA subgroups presented strong biomarker connections regardless of their platelet counts or death risk scores (Figure 9). SCA patients with high reticulocyte counts and high death risk presented a more similar network highlighted by common connections such as the TNF-*α*/IL-1*β*/MIP-1*α* triad, the IFN-*γ*/IL-13, IL-17/IL-4 triad, and the IL-4/IL-2 axes. The SCA patients with high platelet counts displayed a more peculiar network mediated by several positive connections (Figure 9).

## 4. Discussion

The sickle cell anemia is a complex clinical syndrome caused by the presence of HbS and erythrocyte injury that leads to extra- and intravascular hemolysis, inflammation, endothelial dysfunction, and vasculopathy, accompanied by heterocellular leukocyte-platelet-erythrocyte-endothelial adhesive events that trigger vasoocclusive episodes of small and large blood vessels producing acute organ ischemia and reperfusion injury [22]. Many studies have confirmed the association of indirect markers of hemolysis with certain complications of disease. Belini Junior et al. [13] in their study about the severity of Brazilian sickle cell disease patients showed that in all patients the relationship of reticulocytes, hemoglobin, leucocytes, LDH, bilirubin, and HbS biomarkers with severity score was statistically significant; that is, SDC severity is associated with these markers. Our SCA patients at steady state presented an intense reticulocytosis, and when we evaluate the immunologic biomarkers in this condition, the relation between these markers is very similar in the high death risk condition.

The immune abnormalities in SCA can contribute to vasoocclusive crises and increase susceptibility to infection and may potentially explain incidences of impaired magnitude or duration of responses to vaccines. These conditions lead to significant morbidity and/or risk of death in individuals with SCA [9, 13, 27, 31]. In this study, we analyzed cells, receptors, adhesion molecules, cytokines, chemokines, and growth factors of innate and adaptive immunities from SCA patients aiming to better understand the involvement of the immune response in the pathophysiology and morbidity of SCA and thus identify immunological biomarkers that may be related to laboratory conditions and clinical severity of SCA.

Our results explicitly show that patients with SCA at steady state have a high frequency of adaptive immune cells rather than innate immune cells. Although TCD4+ and TCD8+ lymphocytes were significantly decreased in SCA patients compared to the HD group, the frequency of these activated cells was significantly higher in patients compared to the HD group regardless the low and high laboratory and clinical conditions evaluated. The value of T-cell subsets in patients with homozygous SCA is variable [32]. Koffi et al. in 2003 showed that CD8+ T-cells significantly increased and there was no difference between CD4+ T-cells in SCA patients compared to their control group [32]. However, Kaaba and Al-Harbi in 1993 and Vingert et al. in 2015 also showed a lower frequency of TCD4+ and CD8+ T-cells in SCA patients compared to HD individuals [25, 33]. The reduction in the proportion of circulating CD4+ and CD8+ cells was shown to be more profound in the presence of splenic defects, and SCA is invariably accompanied by hyposplenism [27, 32].

The TCD4+/TCD8+ ratio was higher in SCA patients than the HD group, indicating that there is an increased absolute number of TCD4 cells and fewer TCD8 cells in SCA patients (this means a normal ratio). However, a reversal of the normal ratio, that is a relative increase in the number of TCD8+ lymphocytes in SCA patients has been shown by Adedeji in 1985 [34]. Even though these cells were more activated in SCA patients indicating that they are acting on the immune response [25]. Rêgo et al. [35] and Vingert et al. [24] reported in their works of an increase in the regulatory T-cell (CD4+CD25+FoxP3+) frequency in SCD patients compared to the HD group. However, this increase can be related to previous blood transfusion or to the body's attempt to minimize inflammation intrinsic to SCA [24, 35, 36]. Our results show that Tregs appear to be increased in SCA patients compared to HD, but this difference was not significant.

The Mac-1 molecule is very important for mediating the interaction between RBC and adherent leucocytes and promotes vasoocclusion in SDC. It was observed by Chen et al. that Mac-1 deficiency significantly prolonged the survival of SCD mice during and after the experimental procedure [37]. Our results showed that TCD4+CD11b+ may have an effective participation in the process of endothelium adhesion and occlusive vessel crisis regardless of reticulocytosis, thrombocytosis, and death risk in contrast to the TCD8+ lymphocyte that had a low expression of Mac-1 compared to the HD group.

The VLA-4 molecule (CD49d) is the only integrin maintained on the surface of young SS erythrocytes and reticulocytes, which are increased in the peripheral blood of SDC patients and mediate the adhesion of these cells to the endothelium [38]. Canalli et al. [39] demonstrated that VLA-4 in neutrophils, despite its low expression, participates in the *in vitro* adhesion of sickle cell disease neutrophils to endothelial layers under both basal and tumor necrosis factor-*α*-stimulated conditions. Our results showed no difference in VLA-4 expression either of the TCD4+ or TCD8+ lymphocytes in SCA patients compared to the HD group, but it appears to be increased in CD4+ T lymphocytes. However, when we evaluated this marker in the laboratory and clinical conditions of SCA, the high VLA-4 expression in TCD8+ appears to be a selective biomarker for high death condition, as also shown by signature analyses. The higher expression of VLA-4 in TCD8+ lymphocytes increases its adhesion to vascular endothelium, affecting the microenvironment through cytokines and interactions with other cell types and extracellular matrix [40].

Several studies have shown that the proportions of circulating B-cells in SCA are generally unaltered [27, 41, 42]. Venkataraman and Westerman [43] showed no significant changes in the percentage of B-cells in SCA patients at steady state compared to control group individuals; however, they showed that changes in *in vitro* B cell function occur during vasoocclusive pain crises in SCA patients. Unlike the cited data, we observed in our work a significant increase in the frequency of B (CD5-CD19+) in SCA patients at steady state regardless reticulocytosis, thrombocytosis, and death risk conditions compared to HD. Kaaba and Al-Harbi [33] and Bao et al. [44] have also observed increases in B-cells in SCA patients at steady state, and they claim that higher numbers of B lymphocytes are observed in adults with SCA with increased circulatory Th2 cytokines, IL-4 and IL-10. The multiple blood transfusions in SCA patients can induce B-cell differentiation and immunoglobulin secretion and consequently alloimmunization [27].

About innate immunity cells, our results showed a lower frequency of total neutrophil count and no difference in the frequency of total monocyte count compared to healthy donors unlike those of several studies [45–49]. However, in the SCA patients evaluated in our work, the neutrophils appear to be more activated (CD11b+) than HD. Increasing evidence indicates that (activated) neutrophils could play an important role in the initiation and propagation of vasoocclusive processes in SCD [50]. Lard et al. [50] and Kerst et al. [51] observed a similar result with a decrease in neutrophil count that was most likely caused by activated neutrophils due to the high expression of the CD11b molecule and consequently greater adhesion to the vascular endothelium. So, neutrophils may have left the circulation and are therefore underrepresented in the blood. Through our analyses, we observed activated neutrophils expressing CD11b+ at a high frequency in high thrombocytosis and making rare connections with other markers. So, it appears that neutrophils expressing CD11b+ in our SCA patients at steady state are aggregated with platelets. Platelets can also bind to erythrocytes, monocytes, and neutrophils to form aggregates.

Between monocyte subsets, only activated inflammatory monocytes (CD14+CD16+HLA-DR+) were significantly elevated in SCA patients. Singhal et al. [52] showed similar results to our work, which observed a very low count of total monocytes and a high percentage for inflammatory monocytes (CD14+CD16+HLA-DR+). In SDC patients, monocytes presented a significant expression of phosphatidylserine on the surface indicating the probability of the early activation of apoptosis, which could be a possible reason for the low counts of these cells in patients [52]. Annarapu et al. also described in their study that when CD14+ monocytes are engulfed by the Hb-activated platelets, a frequent and characteristic condition of SCA [53], they were transformed into the highly inflamed CD14+CD16+ subtype. This condition may be happening in our SCA patients at steady state.

We observed a high frequency of B1 cells (CD5+CD19+) in SCA patients compared to HD. Human B1 cells (CD5+CD19+) spontaneously secrete IgM and express an Ig repertoire targeted to a narrow range of antigens such as phosphorylcholine and DNA [54]. These cells also promote homeostasis and can modulate the immune response by secreting the anti-inflammatory cytokine IL-10. Natural IgM produced by B1 cells has been shown to promote inflammation by activating the classical complement cascade and cause tissue destruction by binding to epithelial antigens exposed upon ischemia-reperfusion injury [55]. Possibly by this mechanism, B1 lymphocytes are contributing to the pathophysiology of SCA. Furthermore, it was shown by Paglieroni et al. [56] that in SCA patients who had received blood transfusion but who have not received a transfusion within 3 months, there were significant differences in CD5+ B-cell (B1 cell) percentages and absolute numbers compared to healthy never-transfused blood donors.

Among Toll-like receptors, only TLR9 in both neutrophils and monocytes was highly expressed. Pitanga et al. [57] showed a higher expression of *TLR2* and *TLR4* genes and no differences in *TLR9* expression in PBMC of 12 SCA patients at steady state. Although they did not analyze TLR proteins, they believe that the continuous exposure to SS-RBC can affect TLR pathways. Some authors have described that certain conditions, such as elevated IL-4 and IL-10 or an inappropriate concentration of a given stimulus (TLR2 and TLR4 agonists), are not sufficient to induce TLR2 and TLR4 upregulation on the surface of monocytes and neutrophils possibly because the baseline expression of these receptors might be sufficient and an upregulation would only occur after higher stimulus [58–60]. However, we observed through other analyses the high expression of TLR2 in monocytes in high thrombocytosis which appears to be a selective biomarker to high death risk. The network shows TLR2 and TLR4 from neutrophils and monocytes making connections among them and with CD4+CD69+ T-cells.

The circulating cell-free DNA in SCA patients even at steady state can be recognized by TLR9 [61, 62]. Perhaps in our SCA patients at steady state, the hemolysis process and the release of heme are contained, not releasing sufficiently high concentrations to induce upregulation of TLRs 2 and 4, however sufficient to induce TLR9 expression. Besides that, we could observe by analyzing the biomarker network that TLR9 in neutrophils and monocyte makes a positive connection with B-cells both in high reticulocytosis and high death risk. This correlation can indicate that TLR9 stimulation induces maturation of antigen-presenting cells favoring a Th1 immune response and stimulates antigen-independent B-cell proliferation [63, 64].

We are the first to show the profile of classical or myeloid and plasmacytoid DC subsets in SCA patients at steady state. In both subsets, the frequency was low in the SCA patients compared to HD. Urban et al. [65] evaluated cDC and pDC frequencies in children with *α*-thalassemia and the sickle cell trait. They showed a significant reduction of mDC frequency in children homozygous for *α*-thalassemia whether or not they were also carriers for HbS (AA -*α*/-*α* and AS -*α*/*αα*, AS -*α*/-*α*) compared to normal healthy children (AA *αα*/*αα*) [65]. They suggest that the most likely possibilities are reduced mDC production in the bone marrow or chronic activation and enhanced retention of mDCs in the spleen.

Sickle cell disease (SCD) is associated with alterations in immune phenotypes [66], and we decided to evaluate NK and NKT frequencies on SCA patients at steady state. Recently, ischemia-reperfusion injury (IRI) with resultant white cell activation has been implicated as an additional contributor to the pathophysiology of SCD [67]. Our results showed low frequencies of NK and NKT cells in SCA patients at steady state. In ElAlfy et al.'s [66] study, they showed that NK cell frequency was higher in all patients with SDC compared with controls, but the difference was not significant. Furthermore, they showed elevated counts of NK cells with higher levels of IFN-*γ*, so these cells were activated in SCA patients. They suppose that the increased number of NK cells among their patients was associated with a history of frequent vasoocclusive crisis, but they could not compare patients with vasoocclusive crisis and those with stable SCD. Kaplan et al. [68] showed lower NK activity in SCA patients who received repeated blood transfusion compared to normal controls.

The activation of RBCs, leucocytes, platelets, and endothelial cells leads to the increased production of proinflammatory and anti-inflammatory cytokines, which gives SCA characteristics of a chronic inflammatory disease [69]. Our results showed increased levels of Th1 and Th17 cytokine profiles (IFN-*γ*, IL-12, and IL-17) and an inflammatory and regulatory cytokine pattern (IL-1*β*, IL-6, IL-4, and IL-10) in SCA patients at steady state. The chemokines IL-8, IP-10, MIP-1*α*, MIP-1*β*, and RANTES and the growth factors VEGF, FGF-basic, and GM-CSF were higher in SCA patients.

There are a few reports in the literature pertaining to the role of IL-12 and IL-17 in SCA [10, 69]. Unlike our results, Taylor et al. [70] did not observe detectable levels of IL-12 in SCA patients at steady state compared to healthy control subjects; however, as in our study, they observed high levels of IFN-*γ*. In SCA patients from Oman City, the mean serum level of IFN-*γ* was higher in both steady state (95.71 pg/mL) and crisis patients (90.13 pg/mL) compared to control subjects (74.42 pg/mL) [71]. Thus, IL-12 can also negatively affect the development, homeostasis, and function of nTreg cells by limiting IL-2 expression [71]. The immunoregulatory functions of IFN-*γ* are diverse and include the activation of mononuclear phagocytes and neutrophils and the upregulation of class I molecules of the major histocompatibility complex (MHC-1). IL-2 signaling promotes the development of both Th1 and Th2 cell types by inducing the expression of IL-12R*β*2 and IL-4R*α*, respectively [72], and has been reported the suppress Th17 development inhibiting IL-17 production [72].

The role of IL-17 in SCA are not well understood [69]. Corroborating with our results, Vilas-Boas et al. [10] and Pitanga et al. [69] observed higher levels of IL-17 in SCA patients at steady state than in healthy donors. Vilas-Boas et al. still found a positive association between homocysteine and IL-17 and suggest a possible role of homocysteine in the induction of IL-17. We also observed high levels of IL-17 in high thrombocytosis. Bouchnita et al. [73] demonstrated that IL-17 enhanced the level of platelet aggregation from 20% to 45-50% and led to thrombosis and vessel occlusion.

Increased levels of IL-1*β*, IL-6, and TNF-*α* have been reported in serum from patients with SCD [69, 71, 74–77]. Selvaraj et al. [76] showed that monocytes isolated from SCD patients are in a highly activated state as demonstrated by increased gene expression of IL-1*β* as well as TNF-*α* compared with monocytes from healthy individuals. Our results showed detectable levels of TNF-*α* in SCA patients at steady state, but no significant differences. Pathare et al. [71] showed very similar levels of TNF-*α* between SCA patients at steady-state and the control group, but in patients in crisis, the level was higher compared to the control group, despite the nonsignificant difference.

Chemokines contribute to SDC pathogenesis by acting on leucocytes [77]. The IL-8 is involved in both endothelial cell proliferation and angiogenesis produced by several types of cells such neutrophils, macrophages, endothelial cells and induces leucocyte chemotaxis and neutrophil degranulation [69, 77]. Elevated levels of IL-8 are observed in SDC patients during crises and have been proposed to be a vasoocclusion crises predictor [78]. Pathare et al. [71] observed, like our results, significantly high levels of IL-8 in SCA patients at steady state compared to healthy donors, and despite having observed a higher level in patients in crisis, there was no significant difference between steady state and crises. Hazin-Costa et al. [79] also observed high levels of IL-8 in SDC patients at steady state compared to healthy donors.

MIP-1*α* and MIP-1*β* are chemokines with inflammatory properties that are highly related and share 68% identical amino acids [80]. Selvaraj et al. [76] showed a high expression of MIP-1*β* in monocytes activated from SDC patients compared to monocytes from healthy individuals. Wu et al. [81] demonstrated in autoimmune hemolytic anemia that an elevated circulating level of MIP-1*β* indicates a boost in erythroid proliferation and reticulocytosis. It is possible that in SCA, this chemokine can be acting that way. Preclinical studies have further demonstrated that MIP-1*α* is released upon the induction of Th1 responses and by neutrophils and monocytes during monocyte-endothelial cell interactions that serves as an important mechanism in sustaining the recruitment of cells during inflammatory responses [80]. In SCA, MIP-1*α* is possibly also mediating this mechanism. Also, we observed a high level of MIP-1*α* in high thrombocytosis. Klinger et al. [82] showed that MIP-1*α* is present within the *α*-granules of human platelets.

An enhanced expression of MCP-1 was demonstrated in a variety of pathologic conditions associated with inflammation and mononuclear cell infiltration [83]. A higher level of MCP-1 is also associated with the development of polarized Th2 responses [84]. However, our results as well as those of Hazin-Costa et al. [79] did not show a statistical difference in the MCP-1 level between SCA patients at steady state and healthy donors. Although SCA is a chronic inflammatory disease, MCP-1 may be inhibited by the presence of the heme oxygenase 1 (HO-1) enzyme, which is increased in SCA patients [85, 86]. Sickle erythrocytes release large amounts of hemoglobin after hemolysis, and endothelial HO-1 induction may serve to control excessive heme overload [86].

Unlike our results, significant levels of RANTES and IP-10 between SCA patients at steady state and healthy donors was not shown by Hazin-Costa et al.'s results [79]. However, Driss et al. [87] showed elevated serum levels of IP-10 in patients with homozygous sickle cell disease. In the SCA pathophysiology, RANTES still induces the activation of the Gardos channel (Ca-dependent K^+^ channel) in sickle erythrocytes by an increased affinity constant for intracellular Ca^+^ resulting in intracellular depletion of K^+^ and dehydration of sickle red blood cells [88]. Apparently, in SCA, RANTES can be important for the perivascular recruitment of IFN-*γ*-producing T-cells which may affect vascular dysfunction [89].

There are conflicting reports on the role of IL-4 and IL-10 anti-inflammatory cytokines in SCA patients [69]. Pathare et al. [71] and Raghupathy et al. [90] demonstrated significantly higher levels of IL-4 in SCA patients at steady state like our results. Taylor et al. [91] also demonstrated higher levels of IL-4 and IL-10 in SCA patients at steady state, even though Cavalcante et al. [92] did not show significantly different serum levels of IL-10 between steady state SCA patients and control subjects. Musa et al. [14] demonstrated higher levels of IL-4 in SCA patients in vasoocclusive crises than in steady state and control subjects; however, their levels of IL-10 were higher in SCA patients at steady state than in crises and control individuals corroborating our results. The exacerbated inflammatory process within the vascular environment of the SCA patients could be responsible for triggering the compensatory Th2 responses emphasizing the role of an injured endothelium and activated monocytes [93]. The other Th2 cytokines, IL-5 and IL-13, were not elevated in SCA patients at steady state compared to healthy donors unlike what has been demonstrated by Veiga et al. [93]. Possibly, in our SCA patients, the high levels of IL-17 can be affecting IL-5 and IL-13 production.

T-cell homeostasis requires a balance in programs underlying activation and apoptosis with those controlling quiescence and survival. In healthy, T-cell-replete animals, naive CD4 T-cells are sustained by self-peptide and IL-7 signals [94]. We did not demonstrate a significant difference in IL-7 levels between SCA patients and healthy donors. However, in SCA, due to the high levels of activated TCD4^+^ and TCD8^+^ lymphocytes, the IL-7 level maybe be controlled by negative feedback.

Angiogenesis is a highly regulated process and requires coordinated signaling events among a variety of angiogenic factors [95]. Gürkan et al. [96], Solovey et al. [97], and Cao et al. [98] demonstrated higher levels of VEGF and FGF-basic in HbSS patients at steady state corroborating our results and even higher levels in SCA patients with painful vasoocclusive crises. Nevertheless, even platelet activation has also been proposed as a significant contributing factor to the pathogenesis of and outcome of SCD patients [99]. However, they showed that SCA patients during vasoocclusive crises had higher levels of PDGF compared to healthy controls and steady state.

Regarding the colony-stimulating factor, only GM-CSF was higher in SCA patients at steady state compared to healthy donors, while G-CSF was not significantly different. Conran et al. [100] demonstrated that circulating levels of GM-CSF in steady-state patients with SCD were significantly higher than in healthy controls, and G-CSF levels were significantly lower. Besides that, they demonstrated that GM-CSF levels correlated significantly with the numbers of total leukocytes in SCA patients, but G-CSF had a significantly negative correlation. GM-CSF can be responsible, at least in part, for the leukocytosis observed with SCD, and proinflammatory cytokines like IL-1*β*, which are increased in SCD, can be associated with elevated levels of GM-CSF [100].

As discussed above and by the signature and biomarker network analyses, SCA pathophysiology at steady state appears to be controlled by Th1 and Th17 cytokines (IL-12, IFN-*γ*, IL-1*β*, and IL-17), with IL-8, IL-10, and IP-10 as general biomarkers and MIP-1*β* and RANTES making a difference in SCA patients with high death risk. Besides, due to its antiangiogenic activity [101], IL-12 appears to control VEGF regardless of any laboratory and clinical condition, IL-10 appears to control endothelial adhesion by IP-10 correlation, and MIP-1*β* appears to stimulate Th17 response by IL-17 correlation. In high reticulocytosis and death risk, there appears to be a competition of the Th1 and Th17 inflammatory responses with Th2 due to the correlations of TNF-*α*/IL-1*β*/MIP-1*α*, IFN-*γ*/IL-13, IL-17/IL-4, and IL-4/IL-2. In high thrombocytosis, we observed VEGF making several connections with chemokines and cytokines.

In conclusion, our results demonstrated that in SCA pathophysiology at steady state, there is a broad immunological biomarker crosstalk highlighted by TCD4^+^ lymphocytes; TLR2 in monocytes; VLA-4 in TCD8^+^ lymphocytes; Th1, Th17 inflammatory, and IL-10 regulatory cytokines; MIP-1*α*, MIP-1*β*, and IP-10 chemokines; and growth factor VEGF. High levels of MIP-1*β* and RANTES appear to be relevant in a high death risk condition. High reticulocytosis and high death risk conditions present common correlations; there seems to be a balance by Th2 profile. Nevertheless, more studies approaching the same biomarkers in the same patients in crisis or other biomarkers, intracellular signaling pathways, and analysis of gene expression of molecules involved in innate and adaptive immune responses are needed to better understand this complex pathophysiological mechanism of SCA.

## Figures and Tables

**Table 1:** Study population.

Characteristics	Healthy donors HD (*n* = 70)	SCA patients (*n* = 30)	*p* value^∗^
Demographic and epidemiological data			
Gender, male/female (%)	52/18 (74/26)	10/20 (33/67)	—
Age (mean ± SD)	32 ± 11	30 ± 9	—
Residence (%)			
Autazes (AM)		2 (6)	—
Apuí (AM)		1(3)	—
Bacabau (MA)		1(3)	—
Belém (PA)		3 (10)	—
Itacoatiara (AM)		1(3)	—
Manacapuru (AM)		3 (10)	—
Manaquiri (AM)		1 (3)	—
Manaus (AM)		11 (36)	—
Monte Alegre (PA)		1 (3)	—
Óbidos (PA)		2 (6)	—
Oriximiná (PA)		2 (6)	—
Tefé (AM)		1 (3)	—
Ignored		2 (6)	—
Hematological records (median, range)			
Hematocrit (%)	44.75 (35.0-45.1)	24.4 (3.7-33.8)	*p* < 0.0001
Hemoglobin levels (g/dL)	15.15 (11.4-15.6)	7.95 (1.3-11.4)	*p* < 0.0001
White blood cells × 10^6^/mm^3^	6.0 (3.4-6.6)	7.13 (2.5–12.5)	*p* = 0.05
Red blood cells × 10^6^/mm^3^	5.07 (4.1-5.6)	2.47 (0.7-4.5)	*p* < 0.0001
MCV (fL)^∗∗^	87.7 (71.2-92.0)	99.25 (68.6-123.3)	*p* < 0.0001
MCH (pg)^∗∗^	29.8 (24.8-29.7)	31.9 (20.1-42.0)	*p* = 0.0002
CHCM (g/dL)^∗∗^	34.2 (31.0-34.6)	33.6 (30.4-35.2)	*p* = 0.0008
RDW (%)^∗∗^	13.8 (11.9-14.2)	18.2 (15.2-25.6)	*p* < 0.0001
Platelets × 10^6^/mm^3^	246 (100-300)	421 (146.8-859.0)	*p* < 0.0001
MPV (fL)^∗∗^	7.75 (5.8-8.9)	7.6 (6.0-9.5)	*p* = 0.4699
Reticulocytes (%)	—	16.32 (4.2-34.8)	—
Reticulocytes × 10^6^/mm^3^	—	387.45 (163.1-792.6)	—
Signals and symptoms (%)			
Headache	—	19 (63)	—
Joint pain	—	19 (63)	—
Weakness	—	18 (60)	—
Jaundice	—	17 (57)	—
Leg ulcers	—	10 (33)	—
Vasoocclusive crises	—	11 (37)	—
Cholelithiasis	—	8 (27)	—
Splenic sequestration	—	6 (20)	—
Acute thoracic syndrome	—	8 (27)	—
Pulmonar hypertension	—	7 (23)	—
Femur head osteonecrosis	—	5 (17)	—

^∗^Nonparametric test of Mann-Whitney. ^∗∗^Hematimetric indices: MCV—mean corpuscular volume; MCH—mean corpuscular hemoglobin; CHCM—mean corpuscular hemoglobin concentration; RDW—red cell distribution width; MPV—mean platelet volume.

## Data Availability

All data used to support the findings of this study are included within the article.
